# Enhancement of plasmonic coupling on Si metallized with intense femtosecond laser pulses

**DOI:** 10.1038/s41598-023-45968-6

**Published:** 2023-10-27

**Authors:** Mika Tateda, Yuto Iida, Godai Miyaji

**Affiliations:** Faculty of Engineering, Tokyo University of Agriculture and Technology, 2-24-16 Nakacho, Koganei, Tokyo 184-8588 Japan

**Keywords:** Nanophotonics and plasmonics, Ultrafast photonics

## Abstract

Using a pump–probe technique, the reflectivity of a silicon grating surface irradiated with intense femtosecond (fs) laser pulses was measured as a function of the incidence angle and the delay time between pulses. After irradiating the surface with an intense *s*-polarized, 400 nm, 300 fs laser pulse, the reflectivity measured with a weak *p*-polarized, 800 nm, 100 fs laser pulse exhibited an abrupt decrease for an incidence angle of ~ 24°. The depth of the dip was greatest for a delay time of 0.6–10 ps, for which the reflectivity around the dip was highest. The surface was also found to be ablated most strongly for the conditions causing the deepest dip for a delay time of 5–10 ps. Surface plasmon polaritons (SPPs) on silicon metallized by the intense pulse are resonantly excited by the subsequent pulse, and the strong coherent coupling between the subsequent pulse and SPPs excited on the molten Si surface produced by high-density free electrons induces strong surface ablation due to the intense plasmonic near-field. The results clearly show that fs pulses can be used to significantly modulate the nature of nonmetallic materials and could possibly serve as a basic tool for the excitation of SPPs on nonmetallic materials using ultrafast laser–matter interactions.

## Introduction

Ultrafast light–matter interactions can be probed and controlled using a pump–probe technique with ultrashort laser pulses^1–4^. The initial pump pulse changes the state and density of the carriers in the material, leading to a change in light–matter interactions for the subsequent probe pulse. Many experimental and theoretical studies have reported the use of a probe pulse to not only investigate the physical processes involved in ultrafast interactions, such as chemical reactions^1^ and nonlinear optical responses^5,6^, but also to induce particular phenomena such as high-efficiency high-order harmonic generation in gases^7,8^ or on solid surfaces^9,10^, and selective cutting of atomic bonds^11,12^. In particular, in laser material processing, it has been reported that a sharp ablation trace edge and reductions in processing time and energy can be realized by changing the wavelength^13^, pulse duration^14^, polarization^15–17^, delay time^18,19^, and pulse number^20,21^ for the subsequent pulses.

Recently, transient excitation of surface plasmon polaritons (SPPs) with intense femtosecond (fs) laser pulses has been proposed as a mechanism for the formation of laser-induced periodic surface structures (LIPSSs). LIPSS formation has been reported for various kinds of materials, such as dielectrics^22–25^, semiconductors^26–28^, and metals^29–33^. Since the periods range from the order of the laser wavelength *λ* to ~ *λ*/10 or less, this surface phenomenon provides a promising approach for direct laser precision nanoprocessing of materials at a resolution beyond the diffraction limit of light. Recently, it has been applied to functional surfaces such as those used for structural coloration^34^, anti-reflection^35^, superhydrophobicity/superhydrophilicity^36^, friction reduction^37^, and control of cell spreading^38^.

The SPPs can be excited at the interface between metal and dielectric. It has been demonstrated that the intense ultrashort laser pulse irradiation produces electron–hole pairs in dielectrics and metallizes them^39–43^ and predicted that the SPPs could be transiently excited on the metallized dielectric surfaces with intense laser pulses^44–46^. Recently, experimental observations of anomalies in the reflection of intense *p*-polarized 800 nm, 100 fs laser pulses at a nonmetallic material surface with a grating structure have demonstrated that single-shot irradiation by an intense fs laser pulse can transiently produce a high density of free electrons on silicon (Si) surfaces, thereby metallizing the surface, while also exciting SPPs at the interface between the metallized Si surface and air^47,48^. It has also been shown that the intense near-field of the SPPs, the so-called *plasmonic near-field*, can strongly ablate the surface. However, the characteristic properties of SPPs excited on a transiently metallized surface, such as wavelength, amplitude, propagation length, phase, and spatial mode, cannot be identified and controlled using a single-shot irradiation experiment, because the dielectric constant of the Si surface is transiently modulated by irradiation with the intense fs pulse. It is therefore necessary to carry out further studies to elucidate these properties. This would lead to the development of new practical materials nanoprocessing techniques.

In this paper, we report that the coupling between SPPs and an fs pulse can be enhanced on a Si grating surface by adjusting the fluence of the pump pulse and the delay time between the pump and probe fs pulses. In the experiment, we measured the reflectivity of Si surfaces in air as a function of the incidence angle and the delay time between the two pulses, and observed the morphological changes of the surfaces. Preliminary results were presented in^49^. The experimental and calculation results demonstrate that SPPs on Si metallized by an intense fs pulse are resonantly excited by the subsequent fs pulse, and the surface is strongly ablated when the coupling efficiency of the SPPs with an fs pulse at a fluence below the single-shot ablation threshold reaches a maximum on the molten surface produced by high-density electrons.

## Materials and methods

### Experimental setup

**Figure1:**
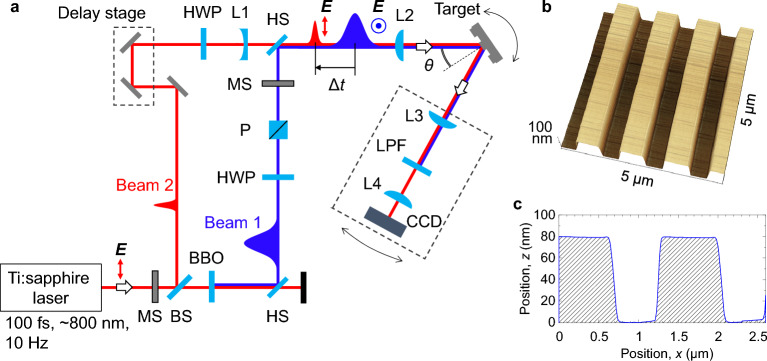
Experimental setup. (**a**) Schematic diagram of optical configuration for time-resolved reflectivity measurements. *E* denotes the polarization direction. The dashed rectangles represent breadboards. The abbreviations are explained in the text. (**b**) SPM image and (**c**) corresponding lateral scan for Si grating surface.

Figure 1a shows a schematic drawing of the optical configuration for measuring the reflectivity *R* of Si grating surfaces using a pump–probe technique. We used linearly polarized, 100 fs laser pulses at a wavelength of *λ* ~ 800 nm produced by a Ti:sapphire chirped-pulse amplification laser system at a repetition rate of 10 Hz. The beam passed through a mechanical shutter (MS in Fig. 1a) and was split into two beams by a beam splitter (BS). The laser pulse transmitted through the BS was frequency-doubled by a 0.5 mm-thick β-BaB_2_O_4_ (BBO) crystal, to ensure production of high-density electrons at the Si surface. The fundamental wave was eliminated using a harmonic separator (HS) and the second harmonic (Beam 1) was transmitted to a pair of a half-wave plate (HWP) and a polarizer (P) to control the pulse energy and produce vertical polarization (*s*-polarization). The pulse duration for Beam 1 was ~ 300 fs, measured by cross-correlation with a BBO crystal for third-harmonic generation. The laser pulse reflected by the BS (Beam 2) was used to excite and observe SPPs. The polarization was set to horizontal (*p*-polarization) using a HWP. The time delay Δ*t* between Beam 1 and 2 was controlled in the range Δ*t* = − 10 to 10 ps using a delay stage. To ensure that Beam 2 uniformly irradiated the target surface area irradiated with the focused Beam 1, we expanded Beam 2 using a lens (L1) with a focal length of *f* = − 220 mm.

The two beams of Beam 1 (*s*-polarization, 400 nm, 300 fs) and Beam 2 (*p*-polarization, 800 nm, 100 fs) were recombined collinearly using an HS and focused onto the target at an incidence angle *θ* using a lens with a focal length of 350 mm (L2). A delay time of Δ*t* = 0 between Beam 1 and 2 was defined as that for which the intensity of the third harmonic was a maximum. Following reflection at the target surface, Beam 1 was damped using a low-pass filter (LPF), while the reflected Beam 2 was expanded with a pair of lenses with focal lengths of *f* = 100 and 500 mm (L3 and L4). Microscopy images and spatial intensity distributions were acquired using a charge-coupled-device (CCD) camera. L3, L4, LPF, and the CCD camera were set on a breadboard that could be rotated around the focal point of Beam 1 at the target surface. The target and the breadboard were set on different rotational stages. When the stage with the target was rotated with *θ*, the other stage with the breadboard was rotated with 2*θ*.

### Materials

As targets, we used a polished *p*-type crystalline Si(100) substrate and a laminar Si grating. This grating was fabricated on the Si substrate by photolithography and dry etching, and had a groove period of *Λ* = 1300 nm, a groove width of 650 nm, and a groove depth of 80 nm, as shown in Fig. 1b,c.

### Experimental method

*R* for the targets was measured for single shots of Beam 1 and 2 for *θ* = 10–60° and Δ*t* = − 10 to 10 ps. At normal incidence, the focal spot sizes for Beam 1 and 2 on the target were, respectively, ~ 40 µm and ~ 650 µm at the 1/*e*^2^ radius. *R* was evaluated from the intensity of the reflected Beam 2 at the central area of Beam 1 with a diameter of ~ 9 μm. The fluence of Beam 1 was *F*_1_ = 50–1000 mJ/cm^2^, while that of Beam 2 was fixed at *F*_2_ < 0.6 mJ/cm^2^, which is much lower than the single-shot ablation threshold of 400 mJ/cm^2^ for an 800 nm fs pulse^47^. With increasing *θ*, the pulse energy for Beam 1 was increased by a factor of cos *θ* to maintain a constant value of *F*_1_ using a pair of HWP and P. The target was moved along the grating surface after each shot so that a fresh part of the target surface was irradiated by the next shot.

To perform ablation experiments by producing an intense plasmonic near-field, the fluence for Beam 2 was increased to *F*_2_ = 100 mJ/cm^2^. This was achieved by changing L1 from a single lens with *f* = − 220 mm to a pair of lenses with *f* = − 1000 and 1500 mm. The spot size for Beam 2 was ~ 55 µm at the 1/*e*^2^ radius at normal incidence.

### Surface morphology observations

The surface morphology of the target was observed using scanning probe microscopy (SPM, SHIMADZU CORPORATION, SPM–9700).

### Calculation method

To calculate the reflectivity and electric field distribution around the grating surface, we used a software-based rigorous coupled-wave analysis (RCWA) method (Synopsys, Inc., DiffractMOD).

## Results

**Figure 2:**
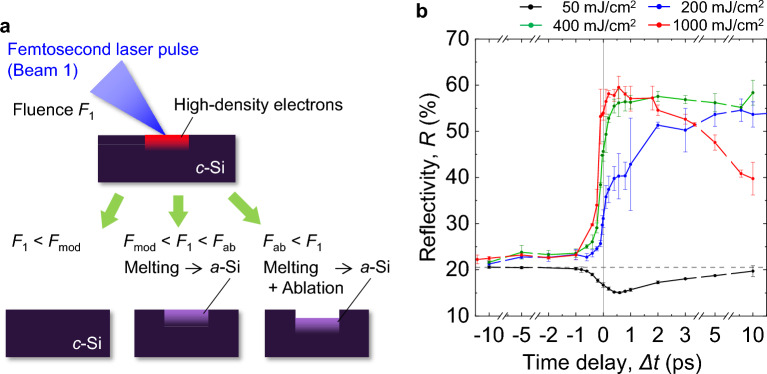
Ultrafast reflectivity dynamics of Si surfaces induced by laser irradiation with various fluences. (**a**) Schematic of Si surface modification for different laser fluences. (**b**) Reflectivity *R* for flat Si substrate at *θ* = 45° measured as function of Δ*t* with Beam 1 (*s*-polarization, 400 nm) for different *F*_1_. The gray dashed line represents the initial value of *R* = 21%.

When a crystalline silicon (*c*-Si) surface is irradiated with an intense femtosecond (fs) laser pulse, free carriers on the surface can be strongly excited to a density of the order of 10^22^ cm^−3^ by the end of the pulse, and the real part of the dielectric constant becomes negative, leading to so-called *metallization*^39–43^. At low fluences, as shown in Fig. 2a, the photoexcited surface reverts back to *c*-Si through a relaxation process, while at high fluences it melts a few picoseconds after pulse irradiation, and ablation occurs within a nanosecond^39–43^. In the latter case, the remaining molten layer is converted to amorphous Si (*a*-Si)^50^. In a preliminary experiment, the surface modification threshold *F*_mod_ and ablation threshold *F*_ab_ for a single shot of a 400 nm, 300 fs laser pulse (Beam 1) were measured in air to be 60 and 420 mJ/cm^2^, respectively, for a flat *c*-Si substrate at normal incidence. To investigate the effect of the Beam-1 fluence *F*_1_, we measured the reflectivity *R* for Si with *F*_1_ = 50 mJ/cm^2^ (lower than *F*_mod_), 200 mJ/cm^2^, 400 mJ/cm^2^ (higher than *F*_mod_ and lower than *F*_ab_), and 1000 mJ/cm^2^ (higher than *F*_ab_).

First, to observe the ultrafast dynamics of the Si surface induced by an intense fs pulse and to determine the *F*_1_ value sufficient to metallize the Si surface, we measured *R* for a flat *c*-Si substrate for an incidence angle of *θ* = 45° and a time delay of Δ*t* = − 10 to 10 ps with a Beam-1 fluence of *F*_1_ = 50, 200, 400, and 1000 mJ/cm^2^. The results are plotted in Fig. 2b. At *F*_1_ = 50 mJ/cm^2^, *R* starts to decrease at Δ*t* ~  − 1 ps, reaches a minimum at Δ*t* ~ 0.5 ps, and then increases monotonically to recover the initial value of *R* = 21%. This indicates that a flat *c*-Si surface is maintained after laser irradiation at *F*_1_ < *F*_mod_. When *F*_1_ is increased to 200 mJ/cm^2^, *R* abruptly increases to ~ 40% at Δ*t* = 0 to 0.5 ps, and then monotonically increases to ~ 54% to become constant for Δ*t* > 5 ps. For *F*_1_ = 400 mJ/cm^2^, *R* increases abruptly for Δ*t* = − 0.5 to 0.5 ps and becomes constant at ~ 57% for Δ*t* > 0.5 ps. With a further increase in *F*_1_ to 1000 mJ/cm^2^, *R* achieves a maximum value of ~ 59% at Δ*t* ~ 0.5 ps and then decreases monotonically for Δ*t* > 0.5 ps.

**Figure 3:**
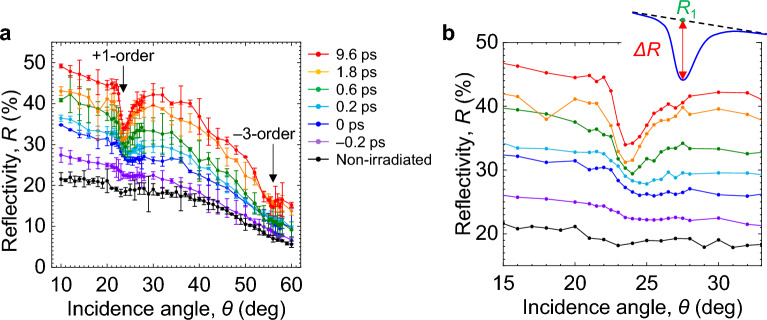
Surface plasmon polaritons on Si gratings resonantly excited by *p*-polarized, 800-nm fs laser pulse. (**a**) Reflectivity *R* as function of *θ* for different Δ*t* for Beam 1 (*s*-polarization, 400 nm) with *F*_1_ = 200 mJ/cm^2^. (**b**) expansion of (**a**). The black data points represent *R* measured in the absence of irradiation. Error bars are omitted in (**b**). The inset shows the definition of *η* = Δ*R*/*R*_1_.

We next measured *R* for a Si grating with *θ* = 10–60°, Δ*t* = − 0.2 to 10 ps, and *F*_1_ = 200, 400, and 1000 mJ/cm^2^. The results obtained for *F*_1_ = 200 mJ/cm^2^ are plotted in Fig. 3. For comparison, *R* in the absence of irradiation is also shown in this figure. The experimental results also show that *R* is higher than the initial value for Δ*t* ≥ − 0.2 ps, indicating that the grating surface is metallized for Δ*t* = − 0.2 to 10 ps. The *R* curves in Fig. 3 exhibit a clear dip at *θ* ~ 24° and a faint dip at *θ* ~ 56°, while those measured with the *s*-polarized Beam 2 do not exhibit a dip, consistent with our previous report^47^. With increasing Δ*t*, the *R* curve near the dip at *θ* ~ 24° becomes steeper and the dip is more pronounced. Because the wavenumber of the SPPs is larger than that of the incident light, to resonantly excite the SPPs with the light, the momentum conservation among the incident light, SPPs, and diffracted light by grating must be satisfied on the surface^51^. Changing the wavenumber of the light on the surface by changing the incidence angle, when the SPPs are resonantly excited at the angle where the momentum conservation is satisfied, the reflectivity abruptly decreases due to the energy transfer of light to SPPs. Based on the momentum conservation among the SPPs, fs pulse, and grating^47^, the dips located at *θ* ~ 24° and ~ 56° represent first- and third-order excitations of the SPPs, respectively. These results show that the SPPs on the Si grating surface transiently metallized by Beam 1 are resonantly excited by Beam 2 at *θ* ~ 24° and ~ 56°, and that the coupling between the SPPs and Beam 2 becomes stronger with increasing Δ*t*.

**Figure 4:**
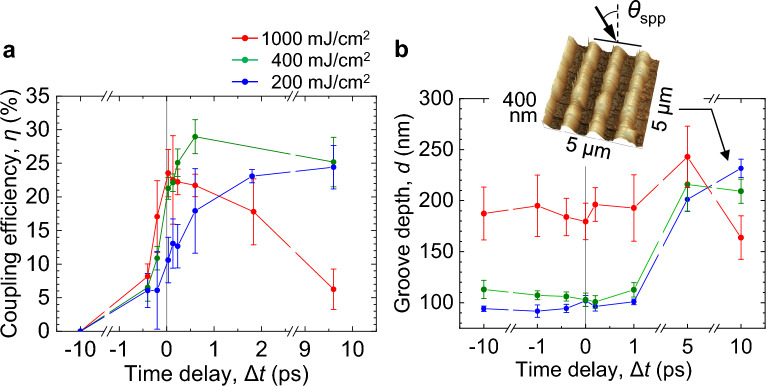
Strong ablation by plasmonic near-fields. (**a**) Coupling efficiency *η* plotted as function of Δ*t* for Beam 1 (*s*-polarization, 400 nm) with different *F*_1_. (**b**) Groove depth *d* for Si grating for different *F*_1_ plotted as function of Δ*t*. The incidence angle of the two beams and the fluence for Beam 2 (*p*-polarization, 800 nm) were *θ* = 23°–24° and *F*_2_ = 100 mJ/cm^2^, respectively. The inset shows an SPM image of the Si grating surface irradiated with *F*_1_ = *F*_2_ = 200 mJ/cm^2^ at Δ*t* = 10 ps and *θ* = 23.0°.

The depth of the dip in the *R* curve represents the coupling efficiency between the SPPs and light, which indicates the ratio of the absorbed SPP power and incident light power at a particular incidence angle *θ*_spp_ for SPP resonance^52,53^. To see how this efficiency changes with Δ*t* and *F*_1_, we evaluated the coupling efficiency *η* from *R* for *F*_1_ = 200, 400, and 1000 mJ/cm^2^ as a function of *θ* and Δ*t*. Here, we defined *η* = Δ*R*/*R*_1_ as the ratio between the decrease Δ*R* in *R* at the dip and *R*_1_ averaged around the dip, as shown in the inset in Fig. 3b^52,53^. The results are shown in Fig. 4a. At *F*_1_ = 200 mJ/cm^2^, *η* increases monotonically from ~ 6 to ~ 25% with increasing Δ*t* > − 0.2 ps. At *F*_1_ = 400 mJ/cm^2^, *η* increases abruptly at Δ*t* ~ 0 ps, to a maximum of ~ 30% at Δ*t* = 0.6 ps, and then remains relatively constant, with only a slight decrease. At *F*_1_ = 1000 mJ/cm^2^, *η* reaches a peak of 25% at Δ*t* ~ 0 ps and decreases monotonically for Δ*t* > 0 ps with increasing Δ*t*. Comparing Fig. 4a with Fig. 2b, the dependence of *η* on Δ*t* for different *F*_1_ values is in good agreement with the dependence of *R* on Δ*t* for the flat Si substrate.

When SPPs are strongly coupled with a fs pulse, a very intense plasmonic near-field is expected to be induced, leading to deep surface ablation. To confirm this, we observed morphological changes of Si grating surfaces irradiated with fs pulses at *θ* = 24°. Figure 4b shows the groove depth *d* plotted as a function of Δ*t*. The ablation depth following a single shot using only Beam 1 with *F*_1_ = 200, 400, and 1000 mJ/cm^2^ was measured to be *d* = 90, 110, and 190 nm, respectively. For *F*_1_ = 200 mJ/cm^2^, *d* increases monotonically with increasing Δ*t* > 1 ps and reaches ~ 240 nm at Δ*t* = 10 ps, and *η* increases monotonically with increasing Δ*t* > –0.2 ps, as seen in Fig. 4a. For *F*_1_ = 400 mJ/cm^2^, *d* increases for Δ*t* > 1 ps and reaches ~ 230 nm at Δ*t* = 5 ps, and *η* reaches a peak at Δ*t* = 0.6 ps and remains relatively constant for Δ*t* = 1–10 ps. For *F*_1_ = 1000 mJ/cm^2^, or higher than *F*_ab_, *d* is 200–240 nm and does not change with increasing Δ*t*, because Beam 1 can directly ablate the surface. These results show that the very intense plasmonic near-field on the surface melted by the high-density free electrons can strongly ablate the surface, and that double fs pulses with a time delay of Δ*t* = 5–10 ps at fluences much smaller than the single-shot ablation threshold *F*_ab_ can cause three times deeper ablation than a single fs pulse.

## Discussion

We used a physical model shown below to discuss the physical processes from the obtained experimental results qualitatively. First, it was assumed that a single shot of focused Beam 1 (*s*-polarization, 400 nm) produced electrons on the Si surface through linear and non-linear optical absorption processes, and a high-electron-density layer was formed on the surface just after the Beam 1 irradiation. It was further assumed that after a few ps, a molten layer was formed on the surface due to energy transfer from electrons to the lattice. In the experiment, the reflectivity after Beam 1 irradiation was measured by irradiating Beam 2 (*p*-polarization, 800 nm). To compare with the experimental results, the dielectric function of Si at *λ* = 800 nm (Beam 2) was calculated with the Drude model, and then the reflectivity of the flat Si substrate and Si grating were determined with a Fresnel equation and the RCWA method, respectively. Here, the change in the dielectric function of Si at high temperature was neglected in this work, because it is much smaller than that with high-density electrons.

### Electrons produced during irradiation by intense femtosecond laser pulse

**Figure 5:**
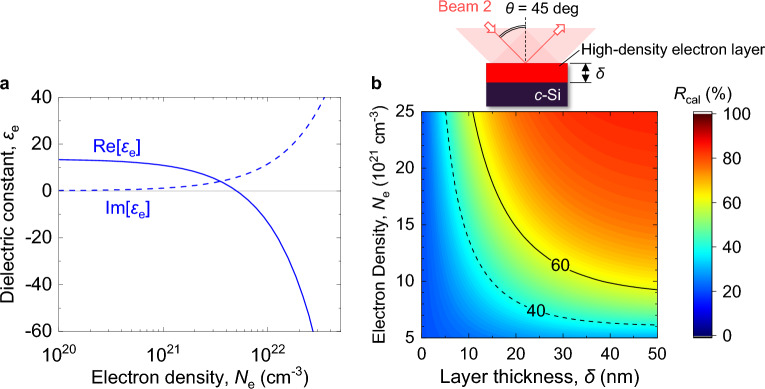
Estimation of electron density and thickness for high-electron-density layer. (**a**) Calculated dielectric constant *ε*_e_ at 800 nm (Beam 2) for Si as function of *N*_e_. The solid and dashed lines represent the real and imaginary parts, respectively. (**b**) Calculated reflectivity *R*_cal_ for flat *c*-Si surface for *p*-polarized, 800-nm light at *θ* = 45° as function of *N*_e_ and *δ*. The inset shows a schematic around the Si surface.

First, we discuss the electron density and thickness of the high-electron-density layer on Si produced by irradiating the focused Beam 1. The complex dielectric constant *ε*_e_ for a Si surface having free electrons with a density of *N*_e_ can be well described using the Drude model^39–41,54^, expressed as

1 $$ \varepsilon_{{\text{e}}} = \varepsilon_{{{\text{Si}}}} \left( {1 - \frac{{N_{{\text{e}}} }}{{N_{{{\text{bf}}}} }}} \right) - \frac{{\omega_{{\text{p}}}^{2} }}{{\omega^{2} + 1/\tau^{2} }} + i\frac{{\omega_{{\text{p}}}^{2} }}{{\omega \tau \left( {\omega^{2} + 1/\tau^{2} } \right) }}, $$

where *ε*_Si_ = 13.5 + *i*0.0384 is the dielectric constant for Si at *λ* = 800 nm of Beam 2^55^, *N*_bf_ = 10^23^ cm^−3^ is the characteristic band capacity of the specific photoexcited regions of the first Brillouin zone in *k*-space associated with band-filling effects^41^, *ω* is the angular frequency of the incident beam in a vacuum, *τ* = 1.1 fs is the Drude damping time for free electrons^41^, *ω*_p_ = [*e*^2^ *N*_e_/(*ε*_0_ m^*^ *m*_e_)]^1/2^ is the plasma frequency, *ε*_0_ is the dielectric constant for the vacuum, *e* is the elementary charge, *m*_e_ is the electron mass, and m^*^ = 0.18 is the effective mass of an electron in metallized Si^41^. Figure 5a shows *ε*_e_ at *λ* = 800 nm plotted as a function of *N*_e_. It can be seen that Re[*ε*_e_] is less than zero for *N*_e_ > 0.5 × 10^22^ cm^−3^, which indicates metallization of the Si. Figure 5b shows the reflectivity *R*_cal_ calculated by the Fresnel equation for a flat *c*-Si substrate with a high-electron-density layer at *λ* = 800 nm and an incidence angle of *θ* = 45°. Here, *R*_cal_ is plotted as a function of *N*_e_ and the layer thickness *δ*. For *δ* > 0 and *N*_e_ > 0.5 × 10^22^ cm^−3^, *R*_cal_ is larger than the initial *R* of 21%. It has been reported that irradiation by a 400 nm, 100 fs laser pulse at *F*_1_ = 60 mJ/cm^2^, which is slightly larger than *F*_mod_, produces a modified layer with a thickness of 17 nm^50^. Here, we assume the presence of a high-electron-density layer with a thickness of *δ* = 10–20 nm produced by Beam 1 with *F*_1_ = 200–1000 mJ/cm^2^ at *θ* = 45°. Based on the measured *R* just after irradiation (Δ*t* = 0.5 ps), as shown in Fig. 2b, the free-electron density *N*_e_ is estimated to be 0.8–1.5 × 10^22^ cm^−3^ for *F*_1_ = 200 mJ/cm^2^, 1.3–2.5 × 10^22^ cm^−3^ for *F*_1_ = 400 mJ/cm^2^, and 1.5–2.7 × 10^22^ cm^−3^ for *F*_1_ = 1000 mJ/cm^2^. These results show that for *F*_1_ ≥ 200 mJ/cm^2^, the real part of *ε*_e_ is negative, indicating metallization of the surface. On the other hand, the slow change in *R* after the pump interaction (Δ*t* ≥ 0.5 ps) for *F*_mod_ < *F*_1_ < *F*_ab_ is caused by many physical processes such as electron–phonon interaction and thermal transportation/diffusion^56^. Here the slow change in *R* is due primarily to energy transfer from free electrons to the lattice, leading to melting of the Si surface^39,56^. The decrease in *R* for *F*_1_ = 1000 mJ/cm^2^ is due to ablation of the surface, because *F*_1_ is much larger than *F*_ab_^57^.

### Excitation of surface plasmon polaritons

Next, we discuss the incidence angle *θ*_spp_ of Beam 2 to excite SPPs on the Si grating. The wavenumber *k*_spp_ of SPPs propagating at the interface between the metallized surface and air is given by

2 $$ k_{{{\text{spp}}}} = k_{0} \sqrt {\frac{{\varepsilon_{{\text{a}}} \varepsilon_{{\text{b}}} }}{{\varepsilon_{{\text{a}}} + \varepsilon_{{\text{b}}} }}} , $$

where *ε*_a_ and *ε*_b_ = 1 are the dielectric constants for the metallized Si and air, respectively, and *k*_0_ = 2π/*λ* is the wavenumber of the incident light with *λ* = 800 nm (Beam 2) in a vacuum. For resonant coupling of SPPs by a grating, the momentum matching equation,

3 $$ {\text{Re}}\left[ {k_{{{\text{spp}}}} } \right] = k_{0} \sin \theta_{{{\text{spp}}}} + q k_{{\text{g}}} , $$

should be satisfied at *θ*_spp_, together with Re[*ε*_a_] < 0, where *q* is an integer, and *k*_g_ = 2π/*Λ* is the wavenumber of a grating with a groove period *Λ*^51^.

From the experimental results for a flat *c*-Si substrate shown in Fig. 2b, assuming that *N*_e_ = 0.8–1.5 × 10^22^ cm^−3^ in the excited layer at Δ*t* ~ 0.5 ps for *F*_1_ = 200 mJ/cm^2^, using *ε*_a_ = *ε*_e_, Eqs. (2)–(3), we can calculate *θ*_spp+1_ = 23.5°–24.4° for *q* =  + 1 and *θ*_spp−3_ = 54.8°–56.4° for *q* = − 3, where *ε*_e_ = –8.7 + *i*8.6 for *N*_e_ = 0.8 × 10^22^ cm^−3^ and *ε*_e_ = − 28 + *i*16 for *N*_e_ = 1.5 × 10^22^ cm^−3^. Assuming that a molten layer is formed at Δ*t* = 2–10 ps, using the dielectric constant for molten Si, *ε*_mSi_ = –22 + *i*43 = ε_a_^58^, Eqs. (2)–(3), we can calculate *θ*_spp+1_ = 22.9° for *q* = + 1 and *θ*_spp–3_ = 57.3° for *q* = − 3. These results agree with the experimental results as shown in Fig. 3.

**Figure 6:**
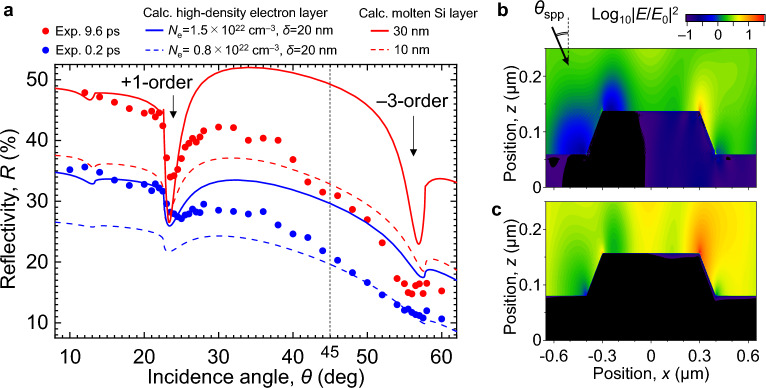
Analysis of experimental and calculation results. (**a**) Comparison of *R* for Si grating irradiated by Beam 1 (*s*-polarization, 400 nm) with *F*_1_ = 200 mJ/cm^2^ for different Δ*t*, and *R*_cal_ for high-electron-density layer and molten Si layer. (**b**) Calculated electric-field intensity distribution produced by incident light at 800 nm (Beam 2) for *θ*_spp_ = 24.0° around high-electron-density layer with *N*_e_ = 1.5 × 10^22^ cm^−3^ and *δ* = 20 nm on Si grating. (**c**) Calculated electric-field intensity distribution produced by incident light at 800 nm (Beam 2) for *θ*_spp_ = 23.0° around molten Si layer with thickness of 30 nm on Si grating.

To investigate SPPs in more detail, the reflectivity of the Si grating and the electric-field intensity distribution around the surface were calculated using the RCWA method for a model target of the Si grating with a high-electron-density layer or a molten Si layer. The calculated reflectivity is compared in Fig. 6a to the measured *R* at Δ*t* = 0.2 ps and 9.6 ps for *F*_1_ = 200 mJ/cm^2^ shown in Fig. 3.

Based on the measured *R* for the flat *c*-Si surface for *θ* = 45° and Δ*t* = 0.2 ps shown in Fig. 2b, and *R*_cal_ shown in Fig. 5b, using *N*_e_ = 0.8 × 10^22^ cm^−3^ and *δ* = 20 nm, the measured and calculated values for *θ* = 45° are similar, as shown in Fig. 6a. In this experiment, *F*_1_ is made independent of *θ* by adjusting the laser energy so that *F*_1_ is constant at each *θ* as described in the Experimental setup section. Actually, because the reflectivity of the target surface for the *s*-polarized Beam 1 increases (that is, the transmittance decreases) as *θ* increases, the density of electrons produced at the target surface and the thickness of the molten layer decrease in increasing *θ*. On the other hand, the physical model used cannot consider that the electron density and the thickness of the molten layer change by this transmittance change, so we separately estimated two different electron densities and two different molten layer thicknesses. Because *N*_e_ for *θ* < 45° is expected to be higher than that for *θ* = 45°, assuming *N*_e_ = 1.5 × 10^22^ cm^−3^ and *δ* = 20 nm, the calculated reflectivity curve is in good agreement with the measured *R* near the dip. Assuming that the surface at Δ*t* = 9.6 ps has been melted by the high-density electrons, the calculated reflectivity for molten Si layer thicknesses of 10 and 30 nm is in good agreement with the experimental results at *θ* = 45° and near the dip, respectively. This clearly indicates that SPPs can be excited at the interface between the high-electron-density layer and air at Δ*t* = 0.2 ps, and at the interface between the molten layer and air at Δ*t* = 9.6 ps.

Finally, we consider the strong ablation caused by the plasmonic near-field shown in Fig. 4b. Figure 6b,c show the calculated results for the electric-field intensity |*E*(*x*, *z*)/*E*_0_|^2^ around the Si grating for *N*_e_ = 1.5 × 10^22^ cm^−3^, *δ* = 20 nm, and a molten layer with a thickness of 30 nm produced by incident light at 800 nm for *θ*_spp_ = 24.0° and 23.0°. Here, *E*_0_ is the incident electric-field amplitude and *E*(*x*, *z*) is the electric-field amplitude at a position (*x*, *z*). In the high-electron-density layer, |*E*(*x*, *z*)/*E*_0_|^2^ = 13 at the right edge of the ridge. However, a value of |*E*(*x*, *z*)/*E*_0_|^2^ = 36 is obtained for the molten layer, which is about three times larger, and an intense electric field is widely distributed at the right edge of the ridge. These results clearly indicate that the electromagnetic energy is concentrated near the surface due to the high plasmon-coupling efficiency, leading to strong nanoscale ablation.

In conclusion, using a pump–probe technique, we investigated the reflectivity of Si grating surfaces to clarify the effect of SPPs excited at the interface between air and a Si surface transiently metallized by an intense fs laser pulse. The results demonstrate that the coupling efficiency of the SPPs with the fs pulse can be enhanced by a factor of three by adjusting the time interval between the two pulses, and that the intense plasmonic near-field generated on the Si surface melted by high-density free electrons can induce strong nanoscale ablation.

## Data Availability

The data that support the findings of this study are available from the corresponding author upon reasonable request.

## References

[1] Zewail AH (2000). Femtochemistry: Atomic-scale dynamics of the chemical bond. J. Phys. Chem. A.

[2] Rossi F, Kuhn T (2002). Theory of ultrafast phenomena in photoexcited semiconductors. Rev. Mod. Phys..

[3] Guo B, Sun J, Lu Y, Jiang L (2019). Ultrafast dynamics observation during femtosecond laser-material interaction. Int. J. Extreme Manuf..

[4] Koya AN, Romanelli M, Kuttruff J, Henriksson N, Stefancu A, Grinblat G, De Andres A, Schnur F, Vanzan M, Marsili M, Rahaman M, Rodríguez AV, Tapani T, Lin H, Dana BD, Lin J, Barbillon G, Zaccaria RP, Brida D, Jariwala D, Veisz L, Cortés E, Corni S, Garoli D, Maccaferri N (2023). Advances in ultrafast plasmonics. Appl. Phys. Rev..

[5] Righini R (1993). Ultrafast optical kerr effect in liquids and solids. Science.

[6] Constant E, Stapelfeldt H, Corkum PB (1996). Observation of enhanced ionization of molecular ions in intense laser fields. Phys. Rev. Lett..

[7] Hay N, Velotta R, Lein M, de Nalda R, Heesel E, Castillejo M, Marangos JP (2002). High-order harmonic generation in laser-aligned molecules. Phys. Rev. A.

[8] Miyazaki K, Kaku M, Miyaji G, Abdurrouf A, Faisal FHM (2005). Field-free alignment of molecules observed with high-order harmonic generation. Phys. Rev. Lett..

[9] Kubodera S, Nagata Y, Akiyama Y, Midorikawa K, Obara M, Tashiro H, Toyoda K (1993). Harmonic generation in laser-produced ions. Phys. Rev. A.

[10] Ganeev RA, Suzuki M, Baba M, Kuroda H (2007). High-order harmonic generation from laser plasma produced by pulses of different duration. Phys. Rev. A.

[11] Kitzler M, Lezius M (2005). Spatial control of recollision wave packets with attosecond precision. Phys. Rev. Lett..

[12] Erattupuzha S, Larimian S, Baltuška A, Xie X, Kitzler M (2016). Two-pulse control over double ionization pathways in CO_2_. J. Chem. Phys..

[13] Zhang J, Sugioka K, Wada S, Tashiro H, Toyoda K (1997). Dual-beam ablation of fused quartz using 266 nm and VUV lasers with different delay-times. Appl. Phys. A Mater. Sci. Process..

[14] Ito Y, Yoshizaki R, Miyamoto N, Sugita N (2018). Ultrafast and precision drilling of glass by selective absorption of fiber-laser pulse into femtosecond-laser-induced filament. Appl. Phys. Lett..

[15] Jia TQ (2005). Formation of nanogratings on the surface of a ZnSe crystal irradiated by femtosecond laser pulses. Phys. Rev. B Condens. Matter Mater. Phys..

[16] Höhm S, Rosenfeld A, Krüger J, Bonse J (2015). Laser-induced periodic surface structures on titanium upon single- and two-color femtosecond double-pulse irradiation. Opt. Express.

[17] Furukawa Y, Sakata R, Konishi K, Ono K, Matsuoka S, Watanabe K, Inoue S, Hashida M, Sakabe S (2016). Demonstration of periodic nanostructure formation with less ablation by double-pulse laser irradiation on titanium. Appl. Phys. Lett..

[18] Semerok A, Dutouquet C (2004). Ultrashort double pulse laser ablation of metals. Thin Solid Films.

[19] Povarnitsyn ME, Itina TE, Khishchenko KV, Levashov PR (2009). Suppression of ablation in femtosecond double-pulse experiments. Phys. Rev. Lett..

[20] Lapczyna M, Chen KP, Herman PR, Tan HW, Marjoribanks RS (1999). Ultra high repetition rate (133 MHz) laser ablation of aluminum with 1.2-Ps pulses. Appl. Phys. A Mater. Sci. Process..

[21] Kerse C, Kalaycloĝ Lu H, Elahi P, Çetin B, Kesim DK, Akçaalan Ö, Yavaş S, Aşlk MD, Öktem B, Hoogland H, Holzwarth R, Ilday FÖ (2016). Ablation-cooled material removal with ultrafast bursts of pulses. Nature.

[22] Bonse J, Sturm H, Schmidt D, Kautek W (2000). Chemical, morphological and accumulation phenomena in ultrashort-pulse laser ablation of TiN in air. Appl. Phys. A Mater. Sci. Process..

[23] Reif J, Costache F, Henyk M, Pandelov SV (2002). Ripples revisited: Non-classical morphology at the bottom of femtosecond laser ablation craters in transparent dielectrics. Appl. Surf. Sci..

[24] Yasumaru N, Miyazaki K, Kiuchi J (2003). Femtosecond-laser-induced nanostructure formed on hard thin films of TiN and DLC. Appl. Phys. A Mater. Sci. Process..

[25] Wu Q, Ma Y, Fang R, Liao Y, Yu Q, Chen X, Wang K (2003). Femtosecond laser-induced periodic surface structure on diamond film. Appl. Phys. Lett..

[26] Borowiec A, Haugen HK (2003). Subwavelength ripple formation on the surfaces of compound semiconductors irradiated with femtosecond laser pulses. Appl. Phys. Lett..

[27] Daminelli G, Krüger J, Kautek W (2004). Femtosecond laser interaction with silicon under water confinement. Thin Solid Films.

[28] Miyaji G, Miyazaki K, Zhang K, Yoshifuji T, Fujita J (2012). Mechanism of femtosecond-laser-induced periodic nanostructure formation on crystalline silicon surface immersed in water. Opt. Express.

[29] Zhao QZ, Malzer S, Wang LJ (2007). Formation of subwavelength periodic structures on tungsten induced by ultrashort laser pulses. Opt. Lett..

[30] Qi L, Nishii K, Namba Y (2009). Regular subwavelength surface structures induced by femtosecond laser pulses on stainless steel. Opt. Lett..

[31] Golosov EV, Emel’yanov VI, Ionin AA, Kolobov YuR, Kudryashov SI, Ligachev AE, Novoselov YuN, Seleznev LV, Sinitsyn DV (2009). Femtosecond laser writing of subwave one-dimensional quasiperiodic nanostructures on a titanium surface. JETP Lett..

[32] Yasumaru N, Sentoku E, Miyazaki K, Kiuchi J (2013). Femtosecond-laser-induced nanostructure formed on nitrided stainless steel. Appl. Surf. Sci..

[33] Miyazaki K, Miyaji G, Inoue T (2015). Nanograting formation on metals in air with interfering femtosecond laser pulses. Appl. Phys. Lett..

[34] Vorobyev AY, Guo C (2008). Colorizing metals with femtosecond laser pulses. Appl. Phys. Lett..

[35] Yang Y, Yang J, Liang C, Wang H (2008). Ultra-broadband enhanced absorption of metal surfaces structured by femtosecond laser pulses. Opt. Express.

[36] Wu B, Zhou M, Li J, Ye X, Li G, Cai L (2009). Superhydrophobic surfaces fabricated by microstructuring of stainless steel using a femtosecond laser. Appl. Surf. Sci..

[37] Yasumaru N, Miyazaki K, Kiuchi J (2008). Control of tribological properties of diamond-like carbon films with femtosecond-laser-induced nanostructuring. Appl. Surf. Sci..

[38] Shinonaga T, Tsukamoto M, Kawa T, Chen P, Nagai A, Hanawa T (2015). Formation of periodic nanostructures using a femtosecond laser to control cell spreading on titanium. Appl. Phys. B Lasers Opt..

[39] Shank CV, Yen R, Hirlimann C (1983). Time-resolved reflectivity measurements of femtosecond-optical-pulse-induced phase transitions in silicon. Phys. Rev. Lett..

[40] Sokolowski-Tinten K, Bialkowski J, Von Der Linde D (1995). Ultrafast laser-induced order-disorder transitions in semiconductors. Phys. Rev. B.

[41] Sokolowski-Tinten K, von der Linde D (2000). Generation of dense electron-hole plasmas in silicon. Phys. Rev. B Condens. Matter Mater. Phys..

[42] Nagai M, Shimano R, Kuwata-Gonokami M (2001). Electron-hole droplet formation in direct-gap semiconductors observed by mid-infrared pump-probe spectroscopy. Phys. Rev. Lett..

[43] Suzuki T, Shimano R (2009). Time-resolved formation of excitons and electron-hole droplets in Si studied using terahertz spectroscopy. Phys. Rev. Lett..

[44] Durach M, Rusina A, Kling MF, Stockman MI (2010). Metallization of nanofilms in strong adiabatic electric fields. Phys. Rev. Lett..

[45] Durach M, Rusina A, Kling MF, Stockman MI (2011). Predicted ultrafast dynamic metallization of dielectric nanofilms by strong single-cycle optical fields. Phys. Rev. Lett..

[46] Derrien TJ-Y, Itina TE, Torres R, Sarnet T, Sentis M (2013). Possible surface plasmon polariton excitation under femtosecond laser irradiation of silicon. J. Appl. Phys..

[47] Miyaji G, Hagiya M, Miyazaki K (2017). Excitation of surface plasmon polaritons on silicon with an intense femtosecond laser pulse. Phys. Rev. B.

[48] Miyaji G, Hagiya M (2019). Reduced damping of surface plasmon polaritons on silicon with intense femtosecond laser pulse. Jpn. J. Appl. Phys..

[49] Iida, Y., Tateda, M., Miyaji, G., Observation of surface plasmon polaritons excited on Si transiently metalized with an intense femtosecond laser pulse, in *2021 Conference on Lasers and Electro-Optics Europe & European Quantum Electronics Conference (CLEO/Europe-EQEC), Munich, Germany*, 1. 10.1109/CLEO/Europe-EQEC52157.2021.9542173 (2021).

[50] Izawa Y, Izawa Y, Setsuhara Y, Hashida M, Fujita M, Sasaki R, Nagai H, Yoshida M (2007). Ultrathin amorphous Si layer formation by femtosecond laser pulse irradiation. Appl. Phys. Lett..

[51] Raether H (1988). Surface Plasmons on Smooth and Rough Surfaces and on Gratings.

[52] Rotenberg N, Caspers JN, Van Driel HM (2009). Tunable ultrafast control of plasmonic coupling to gold films. Phys. Rev. B.

[53] Iqbal T (2017). Coupling efficiency of surface plasmon polaritons: Far- and near-field analyses. Plasmonics.

[54] Danilov PA, Ionin AA, Kudryashov SI, Makarov SV, Rudenko AA, Saltuganov PN, Seleznev LV, Yurovskikh VI, Zayarny DA, Apostolova T (2015). Silicon as a virtual plasmonic material: Acquisition of its transient optical constants and the ultrafast surface plasmon-polariton excitation. J. Exp. Theor. Phys..

[55] Geist J, Palik ED (1997). Handbook of Optical Constants of Solids.

[56] Downer MC, Shank CV (1986). Ultrafast heating of silicon on sapphire by femtosecond optical pulses. Phys. Rev. Lett..

[57] Choi TY, Grigoropoulos CP (2002). Plasma and ablation dynamics in ultrafast laser processing of crystalline silicon. J. Appl. Phys..

[58] Jellison GE, Lowndes DH (1987). Measurements of the optical properties of liquid silicon and germanium using nanosecond time-resolved ellipsometry. Appl. Phys. Lett..

